# Neuropathic Pain and Psychological Morbidity in Patients with Treated Leprosy: A Cross-Sectional Prevalence Study in Mumbai

**DOI:** 10.1371/journal.pntd.0000981

**Published:** 2011-03-08

**Authors:** Estrella Lasry-Levy, Aki Hietaharju, Vivek Pai, Ramaswamy Ganapati, Andrew S. C. Rice, Maija Haanpää, Diana N. J. Lockwood

**Affiliations:** 1 London School of Hygiene & Tropical Medicine, London, United Kingdom; 2 Department of Neurology, Tampere University Hospital, Tampere, Finland; 3 Bombay Leprosy Project, Sion Chunabhatti, Mumbai, India; 4 Department of Anaesthetics, Pain Medicine & Intensive Care, Faculty of Medicine, Imperial College, London, United Kingdom; 5 Department of Neurosurgery, Helsinki University Hospital, Helsinki, Finland; 6 ORTON Rehabilitation, Helsinki, Finland; University of Tennessee, United States of America

## Abstract

**Background:**

Neuropathic pain has been little studied in leprosy. We assessed the prevalence and clinical characteristics of neuropathic pain and the validity of the Douleur Neuropathique 4 questionnaire as a screening tool for neuropathic pain in patients with treated leprosy. The association of neuropathic pain with psychological morbidity was also evaluated.

**Methodology/Principal Findings:**

Adult patients who had completed multi-drug therapy for leprosy were recruited from several Bombay Leprosy Project clinics. Clinical neurological examination, assessment of leprosy affected skin and nerves and pain evaluation were performed for all patients. Patients completed the Douleur Neuropathique 4 and the 12-item General Health Questionnaire to identify neuropathic pain and psychological morbidity.

**Conclusions/Significance:**

One hundred and one patients were recruited, and 22 (21.8%) had neuropathic pain. The main sensory symptoms were numbness (86.4%), tingling (68.2%), hypoesthesia to touch (81.2%) and pinprick (72.7%). Neuropathic pain was associated with nerve enlargement and tenderness, painful skin lesions and with psychological morbidity. The Douleur Neuropathique 4 had a sensitivity of 100% and specificity of 92% in diagnosing neuropathic pain. The Douleur Neuropathique 4 is a simple tool for the screening of neuropathic pain in leprosy patients. Psychological morbidity was detected in 15% of the patients and 41% of the patients with neuropathic pain had psychological morbidity.

## Author Summary

Neuropathic pain has only recently been recognised as a complication of leprosy. We assessed 101 treated leprosy patients in Mumbai and found that 22 of them had neuropathic pain. The pain occurred as numbness 86%, tingling 68%, and decreased sensation to light touch 81%. This pain was significantly associated with nerve enlargement and tenderness, which suggests that ongoing inflammation may be important in causation. A questionnaire-based screening tool (Douleur Neuropathique 4) for detecting neuropathic pain has been developed and validated in other patients groups. We are the first group to have used the DN4 as a screening tool in leprosy patients and found that it worked well, detecting 78% of patients with no inappropriate diagnoses. There is also an increasing recognition that leprosy is associated with psychological morbidity. Neuropathic pain is also associated with psychological morbidity. We also assessed psychological morbidity using the 12-item General Health Questionnaire and found that neuropathic pain and psychological morbidity are associated with leprosy patients. Leprosy patients with neuropathic pain thus have a double hit for psychological morbidity. Clinicians looking after leprosy patients should warn their patients about neuropathic pain and assess their patients for psychological morbidity.

## Introduction

### Leprosy

Leprosy is a chronic granulomatous disease caused by *Mycobacterium leprae* that principally affects skin and peripheral nerves.[1] Leprosy is still present throughout the tropics and sub-tropics. Worldwide 249,007 new cases were registered in 2008 with India registering 134,184.[2] Leprosy affects peripheral nerves causing enlargement, sensory loss and motor weakness, and nerve fibres in the skin causing loss of sensation in affected skin sites. The *M.leprae* infection is treated with multi-drug therapy (MDT) and all patients receive either dual or triple drug therapy for up to 12 months. MDT is highly effective with a relapse rate of 1%. New nerve damage is treated with steroid therapy, but only about 50% of patients will have improvement in nerve function after a course of steroid treatment.[3] Leprosy is also complicated by further episodes of inflammation affecting skin and nerves. These may be Type 1 reactions associated with delayed type hypersensitivity which cause inflammation affecting skin and nerves. Type 2 or erythema nodosum leprosum (ENL) reactions are associated with immune complex deposition and systemic inflammation is seen with involvement of skin, nerves, eyes, bones and testes. Inflammation of nerves is prominent in leprosy pathology.[4]

### Neuropathic pain

Neuropathic pain is defined as “pain arising as a direct consequence of a lesion or disease affecting the nervous system”.[5] This may be due to nerve damage at a peripheral or central level. Neuropathic pain typically persists after the primary cause has resolved. Neuropathic pain is characterised by positive and negative symptoms including pain, hypoesthesia to touch, tingling, electric shocks and pins and needles. The diagnosis of neuropathic pain rests on clinical judgement, a relevant clinical history and clinical neurological examination.[6] The patients should complain of pain that is not generated by a stimulus and it must be in one or more regions related to affected nerves (anatomic plausibility). A diagnosis of probable neuropathic pain can be made if 1) clinical examination shows positive or negative sensory signs confined to innervation territory of the lesioned nervous structure or if 2) diagnostic tests can confirm lesion or disease explaining the neuropathic pain. If both of these criteria are met, the diagnosis of definite neuropathic pain can be made.[7]

Several diagnostic questionnaires have been developed to alert the clinician about the possibility of neuropathic pain, for example the Douleur Neuropathique 4 (DN4). The DN4 consists of seven items related to sensory descriptors and three related to signs which require a simple clinical examination.[8] It examines the positive and negative sensory symptoms of neuropathic pain, such as evoked pain and hypoesthesia to touch. The DN4 has a sensitivity of 83% and specificity of 90%.[8]

### Neuropathic pain in leprosy

Little is known about neuropathic pain in leprosy patients. 26.4% of 358 newly presenting leprosy patients from a referral centre in Brazil had neuropathic pain.[9] 29% of 96 Ethiopian patients who had been treated for leprosy more than 10 years earlier had neuropathic pain, which was “severe” in 43%.[10]

The clinical presentation of neuropathic pain in leprosy patients can be continuous or intermittent and occur at a single or multiple locations. Studies in India and Brazil found most patients with neuropathic pain had a “glove and stocking” symptom distribution, characteristic of polyneuropathy [9], [11], and in the Indian study neuropathic pain was associated with nerve tenderness.[11]

### Psychological morbidity in leprosy and neuropathic pain

Several studies have shown increased prevalence of psychological morbidity in patients with leprosy. In some series, up to 65% of patients have psychological morbidity, depression being the most common.[12], [13], [14] Chronic neuropathic pain has also been associated with psychological and quality of life comorbidities, including circadian rhythm disturbance, anxiety and depression.[15] So leprosy and neuropathic pain may have separate and perhaps synergistic contributions to psychological morbidity.

Non-psychotic mental disorders are defined in ICD-9 as “disorders in which the symptoms are distressing to the individual and recognized by him or her as being unacceptable”.[16] The General Health Questionnaire-12 (GHQ-12) is used to detect the presence of non-psychotic psychological morbidity. It is a self-reporting questionnaire intended for use in a primary health care setting.[17] It consists of 12 questions, asking patients about their general level of happiness, experience of depressive and anxiety symptoms, and sleep disturbance over the last four weeks.[15]

This cross-sectional study was designed to assess the prevalence and characteristics of neuropathic pain in leprosy patients who have completed MDT. From previous studies [11], [18], we expected a prevalence of approximately 15%, because the patients were recruited at a referral centre. This would be higher than in the general population [19], [20], and lower than in patients with diseases in which neuropathic pain is the main feature, such as diabetes or stroke.[21], [22]. Neuropathic pain was assesed using clincial examination. We also assesd pain using the DN4 questionnaire. Psychological morbidity was assesed using the GHQ-12. With this study design we were able to asses the prevalence of neuropathic pain. We also tested the utility of the DN4 as a screeening tool for neuropathic pain in this group. We also predicted that we would find increased psychological morbidity in the patients with neuropathic pain.

## Methods

### Patients

Between July and August 2008, we enrolled 101 patients (73 male [72.3%]; 28 female [27.7%]) from Bombay Leprosy Project clinics in the state of Maharashtra, India. Every patient (estimate n = 150) over the age of 16 who attended the clinics during the study period and had taken a full course of MDT was invited to participate. We did not collect reasons for non participation in the study as we should have done. This sample size was used because we estimated that we would detect about 20 patients with neuropathic pain.

### Diagnosis of leprosy

Leprosy was diagnosed when a patient had one of the following: skin lesions typical for leprosy; and/or thickened peripheral nerves; and/or acid fast bacilli on slit skin smears.[23] Patients with leprosy were then classified using the 1998 WHO classification in which patients are classified as paucibacillary (PB) if they have up to five skin lesions and as multibacillary (MB) if they have five or more skin lesions.[23] The Ridley-Jopling classification was made on the basis of the clinical features and bacterial index.[24]

#### Case definition for neuropathic pain

Patients were defined as having neuropathic pain if distribution of pain was neuroanatomically plausible, confirmatory tests of neurological examination demonstrated positive or negative sensory signs confined to the innervation territory of the affected peripheral nerves, and pain was causally associated with having had leprosy.[7]

### Procedures

**Figure 1:**
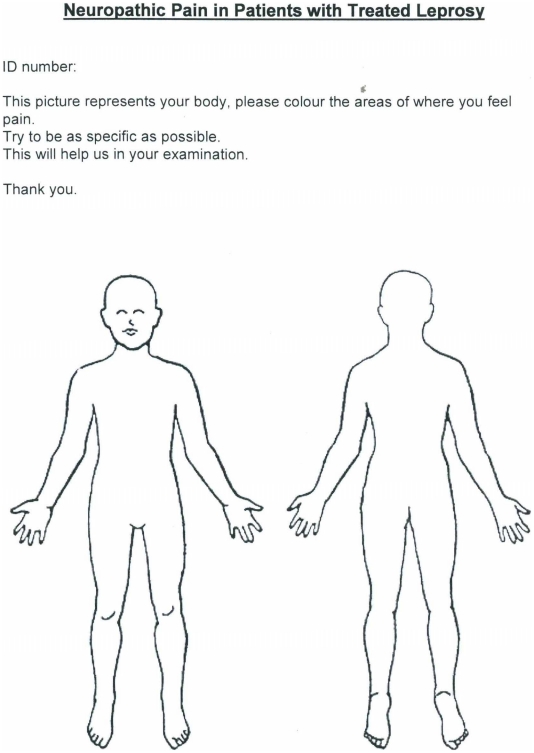
Body chart.

All patients were given a blank body chart (Figure 1) and asked to draw in any areas of pain. The clinician used the same chart to draw patches or skin lesions. A clinical history was then taken. Data was collected on past medical history, diagnosis of leprosy, leprosy antibiotic treatment, leprosy reactions (Type 1, ENL, Neuritis), past and current treatments for leprosy reactions, including corticosteroids, thalidomide, azathioprine and chloroquine. This was followed by a clinical evaluation and the patients completed the DN4 and GHQ-12 questionnaires. The DN4 was translated into Hindi and Marathi, and the GHQ-12 was translated to Hindi and simultaneously translated to Marathi when needed.

### Nerve evaluation

Nerve enlargement and tenderness was clinically evaluated by palpation of the main peripheral nerves (great auricular, ulnar, median, lateral popliteal and posterior tibial), and defined as present or absent. Motor function was tested in the following nerves by assessing the muscle they supply: facial nerve (orbicularis oculi) ulnar nerve (abductor digiti minimi), median nerve (opponens pollicis) and common peroneal nerve (extensor hallucis longus). Sensory testing was done using a series of Semmes-Weinstein monofilaments (MF) (0.05 gm / 0.2 gm / 2 gm / 4 gm / 10 gm / 300 gm) to assess punctate static light touch perception in skin areas supplied by the ulnar, median and posterior tibial nerves.[25] Perception was assessed using a Yes/No response. Sensory impairment was defined as present when the MF threshold increased by three or more levels (filaments) on any site, or two levels on one site and at least one level on another site, or one level on three or more sites for one nerve [26], with a lower limit of 0.2 gm MF perception on the hands, face and leprosy skin lesions, and for 2 gm on the foot.

### Disability assessment

The WHO disability criteria, which has three levels, 0, 1 and 2, was used to document disability. Zero is scored when no disability is present, 1 when loss of sensation in hand or foot is present, and 2 when there is visible damage or disability including deformity, lagophthalmos or hand or foot ulcer.[25]

### Neuropathic pain assessment and questionnaires

Both DN4 and GHQ-12 questionnaires were completed by the patient, with the clinician asking the questions since many patients were not functionally literate. When completing the DN4 questionnaire, if the patient had any of the symptoms, the area in which s/he had it was recorded beside each symptom. In these cases a clinical assessment of the area was undertaken, evaluating ‘hypoesthesia to touch’ by applying light touch with finger on the painful area and a non-painful area simultaneously, and ‘hypoesthesia to pinprick’ using the 300 g MF. Dynamic mechanical allodynia was evaluated using a brush on the painful area and patients responded Yes/No to the evoking of pain.

For the GHQ-12, the patients were asked how they had felt during the past four weeks. If they had any symptoms, they were asked what cause they attributed to them. Interpretation of the answers is based on a four point response scale scored using a bimodal method (symptom present: ‘not at all’  =  0, ‘same as usual’  =  0, ‘more than usual’  =  1 and ‘much more than usual’  =  1). A score of four or more indicates the presence of a disorder, without diagnosing the exact pathology.[17]

### Ethical approval

Ethical approval was received from the London School of Hygiene and Tropical Medicine Ethics Committee prior to commencing the study. Ethical approval in India was received from the Bombay Leprosy Project Managing Committee.

### Informed consent

On recruitment, patients were given an explanation of the study, which was translated to Hindi or Marathi when needed before being invited to sign the consent form which was also in Hindi and Marathi. No patient was paid to participate in the study.

### Statistical analysis

A database was created using a Microsoft Excel spreadsheet, and analysed using Stata 10.1. A χ2 test was then used comparing all the variables to the outcome variables neuropathic pain, psychological morbidity, and presence of disability. For those variables with a significant p value a Mantel-Haenszel test was performed (Table 1). Missing data related mainly to aspects of the patients medical history.

**Table 1:** Patient characteristics according to presence or absence of neuropathic pain.

		Patients with neuropathic pain (*) N = 22	Patients without neuropathic pain (**) N = 79	TOTAL (***) N = 101
**Sex**	Male	14 (63.6%)	59 (74.7%)	73 (72.3%)
	Female	8 (36.4%)	20 (25.3%)	28 (27.7%)
**Mean age**		39.91 years	39.19 years	39.34 years
**WHO classification**	MB	20 (90.9%)	74 (93.7%)	94 (93.1%)
	PB	2 (9.2%)	5 (6.3%)	7 (6.9%)
**Leprosy reaction present**		15 (68.2%)	51 (64.6%)	66 (65.4%)
**Type of reaction**	Type 1 reaction	7 (31.8%)	17 (21.6%)	24 (23.8%)
	ENL	7 (31.8%)	28 (35.4%)	35 (34.7%)
	Neuritis	1 (4.5%)	2 (2.5%)	3 (3.0%)
	Silent neuritis	0	4 (5.1%)	4 (4.0%)
	No reaction	7 (31.8%)	28 (35.4%)	34 (33.7%)
**Other treatments**	Prednisolone	18 (81.8%)	48 (60.8%)	66 (65.4%)
	Thalidomide	3 (13.6%)	23 (29.1%)	26 (25.7%)
	Chloroquine	3 (13.6%)	16 (20.3%)	19 (18.8%)
	Azathioprine	1 (4.5%)	9 (11.4%)	10 (9.9%)
**Presence of patches**		16 (72.7%)	53 (67.1%)	69 (68.3%)
**Painful patches**		9 (40.9%)	15 (19.0%)	24 (23.8%)
**Thickened nerves**		15 (68.2%)	31 (39.2%)	46 (45.5%)
**Tender nerves**		10 (45.5%)	9 (11.4%)	19 (18.8%)
**Sensory impairment**		21 (95.5%)	60 (76.0%)	81 (80.2%)
**Motor impairment**		11 (50.0%)	35 (44.3%)	46 (45.5%)
**DN4 score**	<4 / 10	0	73 (92.4%)	73 (72.3%)
	≥4 / 10	22 (100.0%)	6 (7.6%)	28 (27.7%)
**Psychological morbidity**		9 (40.9%)	6 (7.6%)	15 (14.9%)
**Disability (WHO score of 1 or 2)**		11 (50.0%)	33 (41.8%)	44 (43.6%)

*% of total patients with neuropathic pain (22);

**% of total patients without neuropathic pain (79);

***% of total sample population (101).

## Results

One hundred and one patients were recruited (73 male [72.3%]; 28 female [27.7%]), age range 17 to 89 years (mean 39.3, sd±15.89), of whom 22 (21.8%) had neuropathic pain. Ninety four patients (93%) had MB leprosy, and 7 (7%) had PB leprosy. Most patients (73%) had been diagnosed and treated for leprosy between 2001 and 2007; 20 between 1991 and 2000. During the period of recruitment about 150 patients were reviewed from urban and rural areas in the Bombay Leprosy Project clinics.

### Clinical findings (Table 1)

Sixty nine patients (68.3%) had ongoing skin involvement with patches, nodules, ulcers or infiltration. Twenty four (23.8%) had painful skin lesions, of whom 9 (37.5%) had neuropathic pain. Forty six patients (45.5%) had nerve enlargement and 19 (18.8%) had nerve tenderness. Nerve enlargement was present in one nerve only in 14 patients (30.4%) and in multiple nerves for 32 (69.6%). Nerve enlargement was found most often in the ulnar nerve (65.22%), then the lateral popliteal (43.5%), posterior tibial (39.1%) and greater auricular (37.0%) nerves. Nerve tenderness was most commonly elicited in the ulnar nerve (68.4%), followed by the posterior tibial (57.9%), lateral popliteal (31.6%) and great auricular (15.8%) nerves.

Ulnar nerve motor impairment was detected in 35 patients (34.7%). Combining motor and sensory testing, 65 patients had ulnar nerve impairment (64.4%) and 42 (41.6%) median nerve impairment. Seventy patients (69.3%) had plantar sensory impairment. Sensory testing detected impairment in the areas supplied by the lateral and medial plantar (69.3%), ulnar (45.5%), median (38.6%) and trigeminal (6.9%) nerves.

### Neuropathic pain (Table 2)

**Table 2:** Features of neuropathic pain in patients.

Limb affected	Patient	Age / Gender	Distribution of neuropathic pain	Distribution of nociceptive pain	Thickened nerves	Tender nerves	DN4 score
**Upper**	78	28/M	Ulnar nerves bilaterally	None	Right ulnar nerve, posterior tibial nerves bilaterally	Ulnar and posterior tibial nerves bilaterally	4
	80	50/M	Ulnar nerves bilaterally	None	Left great auricular nerve, ulnar nerves bilaterally, right common peroneal nerve	Left great auricular nerve, ulnar nerves bilaterally, right common peroneal nerve	5 (?)
	84	75/M	Right ulnar nerve	None	Ulnar nerves bilaterally, left posterior tibial nerve	Ulnar nerves bilaterally, left posterior tibial nerve	6
	87	18/F	Right ulnar nerve	None	None	Right ulnar nerve	5
	101	21/F	Right thumb	None	Left ulnar nerve, posterior tibial nerves bilaterally	None	4
**Lower**	1	49/M	Right peroneal nerve and right plantar nerve	None	None	None	5
	17	50/M	Sural and peroneal nerves bilaterally	Elbows	Right ulnar and great auricular nerves	None	5
	20	19/F	Left peroneal nerve	Left arm and hip	None	None	5
	24	54/F	Soles of both feet	None	None	None	6
	26	28/M	Left peroneal nerve, soles of both feet	Left ankle and knee	None	None	7
	41	65/M	Both calves, right sole, left heel	None	Common peroneal nerves bilaterally	Common peroneal nerves bilaterally	6
	43	26/M	Both soles	None	Ulnar and posterior tibial nerves bilaterally, left common peroneal nerve	Ulnar and posterior tibial nerves bilaterally	6
	50	43/F	Right deep peroneal nerve	Right toe	Right posterior tibial nerve	Ulnar nerves bilaterally, right posterior tibial nerve	7
	76	25/M	1st and 2nd toes of left foot	None	Great auricular, ulnar and common peroneal nerves bilaterally, right tibial posterior nerve	None	4
**Both**	3	33/M	Right ulnar nerve and distal symmetric polyneuropathy	None	Right great auricular nerve	None	6
	29	52/M	Right auricular nerve, dorsum of right foot, sole of left foot	None	Right ulnar and posterior tibial nerve	Right posterior tibial nerve	8
	49	45/F	Distal symmetric polyneuropathy	None	None	None	5
	56	43/M	1st and 2nd branches of left trigeminal nerve, left big toe	None	Great auricular nerves bilaterally	None	6
	62	30/M	Distal symmetric polyneuropathy	None	Right ulnar nerve	Right ulnar nerve, left posterior tibial nerve	4
	66	43/F	Distal symmetric polyneuropathy	None	Ulnar nerves bilaterally	Right ulnar nerve, common peroneal and posterior tibial nerves bilaterally	5
	94	28/M	Ulnar nerves bilaterally, both soles and right foot	None	Ulnar and posterior tibial nerves bilaterally, left common peroneal nerve	None	4
	96	46/F	Peroneal nerves bilaterally, right index finger	None	None	None	4

Twenty two patients had neuropathic pain (21.8%) by the clinical definition, and Table 2 gives the features of the pain. Neuropathic pain occurred in areas supplied by ulnar (6), lateral popliteal (6), plantar medial and lateral (4), posterior tibial (2), sural (1), trigeminal (1), tibial anterior (1) and median (1) nerves respectively. Eleven patients had pain in one limb only and 11 had pain affecting both limbs, either arms or legs. Twenty one patients with neuropathic pain had sensory impairment (95.5%) and 11 (50.0%) had motor impairment. Nine (40.9%) had painful leprosy skin lesions in addition to the areas of neuropathic pain associated with nerve trunks or cutaneous nerves.

Patients described sensory symptoms as numbness (19), tingling (15), burning sensation (13), electric shocks (11), pins and needles (10), painful cold (6) and three had itching and these symptoms overlapped in individual patients. Eighteen patients had hypoesthesia to touch in the painful area, 16 had hypoesthesia to pinprick, and one had allodynia.

Twenty eight (27.7%) patients scored 4 or more on the DN4 questionnaire whilst 22 had neuropathic pain by the clinical definition. None of the patients clinically diagnosed with neuropathic pain scored less than 4 on the DN4. These six patients experienced some of the symptoms listed in the questionnaire occasionally in a certain area, but without pain or discomfort. One patient had pain with no anatomical plausibility. In this group the DN4 used for screening for neuropathic pain had a sensitivity of 78.6% and a specificity of 100%.

### Psychological morbidity

Fifteen (14.9%) patients had psychological morbidity, mainly anxiety, mild or incipient depression, which they associated with having leprosy. Forty one percent of the patients with neuropathic pain had psychological morbidity. Some patients who scored less than 4 on the GHQ-12 explained that although they had some symptoms, such as lack of sleep or increased strain, they felt that these were not associated with their leprosy.

### Disability

Forty four patients (43.6%) had disability, eight had grade 1 (7.9%), and 36 grade 2 (35.6%). Of the 22 patients with neuropathic pain, 11 (50%) had disability grade 0, five had grade 1 (22.7%) and six grade 2 (27.3%).

Neuropathic pain is significantly associated with psychological morbidity (p = 0.0001), nerve enlargement (p = 0.016), nerve tenderness (p = 0.0003), trigeminal nerve impairment (p = 0.0194), and painful skin patches (p = 0.0335) (Table 3). No significant association was found between neuropathic pain and sex, age, type of leprosy (WHO or Ridley-Jopling), presence or type of reaction, type of treatment for leprosy, other treatments (prednisolone, thalidomide, chloroquine and azathioprine) or disability. Psychological morbidity was also significantly associated with nerve tenderness (p = 0.024, OR = 3.74, 95% CI 1.09–12.77).

**Table 3:** Analysis of association of neuropathic pain with key leprosy clinical features.

Variable		P	OR	95% CI
**Sensory impairment**		**0.0434**	**6.65**	**0.79–55.71**
	Mild	0.4953	1.58	0.42–6.00
	Moderate	0.4294	1.90	0.38–9.59
	Severe	0.9657	1.03	0.24–4.45
**Motor impairment**		0.6368	1.26	0.48–3.26
**Psychological morbidity**		0.0001	8.42	2.30–30.79
**Thickened nerves**		0.0165	3.32	1.17–9.39
**Tender nerves**		0.0003	6.48	2.01–20.95
**Facial sensory impairment**		0.0194	5.62	1.02–28.90
**Presence of disability**		0.4934	1.39	0.70–6.80

Analyses done with The Mantel-Haensel test.

## Discussion

The study found a high prevalence of neuropathic pain (21.8%) among patients with treated leprosy at a referral centre in India. Neuropathic pain was associated with nerve enlargement and tenderness, and painful skin patches. Psychological morbidity was also significantly associated with neuropathic pain. In all patients, the diagnosis of definite neuropathic pain could be made because they had an established diagnosis of leprosy, and also positive and negative sensory signs on clinical neurologic examination correlating with the peripheral nerves that are affected in leprosy.[7]

The prevalence of neuropathic pain in treated leprosy patients was higher than expected from clinical practice, but similar to that found in patients with diabetic neuropathy in India (26.1%).[27] Comparing with other infectious disease aetiologies, post herpetic neuralgia has been reported in 13.4% of general practice patients in the UK [28] whilst HIV sensory neuropathy affects 42% of patients in Australia.[29] Patients and health care workers probably pay more attention to the symptoms of leprosy itself than to the diagnosis of pain, and appropriate tools and training for diagnosis are not always available.

Our study design, a cross sectional prevalence study, means that the patients in this study are typical of patients who attend leprosy clinics with post-treatment complications. Thus we had a bias towards patients with MB leprosy which is not surprising because patients with MB leprosy are at a higher risk of developing nerve damage and reactions.[26] This also explains why 65% of patients in this study had experienced a past or current reaction. Future prospective studies could examine the effect of reactions on neuropathic pain better and also the association with PB leprosy. However, our findings still reflect the magnitude of the problem amongst patients attending leprosy clinics and show the need for “care after cure” programmes and research in this field. In 2007, a research workshop recommended studies on the epidemiology of neuropathic pain in leprosy as a research priority.[30]

We found a significant association between neuropathic pain and nerve enlargement. Nerve enlargement is one of the diagnostic criteria for leprosy and is present in significant numbers of new patients. However, the persistence of nerve enlargement after treatment suggests that there is ongoing disease in the peripheral nerves of our patients with neuropathic pain. The association with nerve tenderness is also important because this is evidence of ongoing inflammation in the patients with neuropathic pain. A previous study by Lund *et al* in 17 patients with neuropathic pain attending a leprosy clinic in Hyderabad found nerve tenderness in multiple nerves.[11] Nerve biopsies were taken from nine patients and nearly all of them showed neural inflammation and also fibrosis. The association with tender nerves indicates that acute nerve damage may result in chronic injury. This again highlights the importance of early diagnosis in leprosy so that nerve inflammation can be reduced. Further research on this could include nerve biopsies or nerve ultrasound, when available, to detect changes in nerve structure due to inflammation or fibrosis.[31], [32] However, it is also possible that leprosy patients who have reactions and associated acute nerve inflammation might at that point be experiencing the single episode pathogenesis that patients with post herpetic neuralgia experience. Further studies will need to delineate the contributions of leprosy-associated acute and chronic inflammation to leprosy neuropathic pain.

The relationship of neuropathic pain with painful skin patches will require further histopathological studies such as skin biopsy evaluation of intra-epidermal nerve fibre assessment. The patients in this study are probably typical of patients who attend leprosy clinics with late complications. Sixty-six percent of patients had ongoing or a history of leprosy reactions and their medication, including steroids, thalidomide and other immunosuppressants, reflects this. It is interesting that similar numbers of patients with and without pain are taking steroids. It is interesting that we did not find an association between use of thalidomide and neuropathic pain, since painful neuropathy is a well recognised adverse affect of thalidomide.[33] Thalidomide has not been reported to produce neurological sequelae in leprosy patients taking it for ENL although there have not been any well designed prospective studies of this.[34] Those patients who are discharged from the leprosy clinics as “cured” may not wish to access these health facilities in the future.

The DN4 questionnaire performed well in this group and the resource poor field setting with a sensitivity of 100% and a specificity of 92% which compares well with other analyses.[8] In Turkish patients the DN4 had a sensitivity of 95% and a specificity of 96.6%.[35] None of the patients who were clinically diagnosed as having neuropathic pain scored less than 4 on the DN4 questionnaire, although six patients with a score of 4 or more were not diagnosed as having neuropathic pain. The DN4 was easy to implement and understand both for the clinician and the patient, both in English, and its translations to Hindi and Marathi. The patients clearly described the symptoms listed in the questionnaire, independent of their educational level.

Leprosy patients have a large burden of post-disease complications which are not easy to detect since the patient will not always access leprosy clinics, and health workers at general health facilities may not be able to diagnose and treat them. Neuropathic pain is a chronic complication that could possibly be prevented if nerve damage is detected at an early stage and appropriate treatment with corticosteroids is started. Although steroid treatment of even early nerve damage has a variable efficacy with 50–65% of patients responding with improved nerve function after a course of steroid treatment.[36]

The association of neuropathic pain with psychological morbidity increases the importance of neuropathic pain, not only as a physical symptom, but as a possible cause of psychiatric co-morbidity. A study on HIV associated neuropathy in South East Asia found that 20% of patients had sensory neuropathy and 36% evidence of depression.[37] Reverse causality cannot be fully discarded, since psychiatric patients have been shown to have a decreased threshold for pain.[22] This finding also highlights the importance of including mental state and depression evaluations in studies of neuropathic pain, especially if tricyclic antidepressants were to be tested for efficacy in treating neuropathic pain.

No randomised controlled trials of analgesic treatment for neuropathic pain in leprosy or even well designed exploratory studies have been reported.[38] A study in Brazil set the guidelines for treatment, using drugs which have been tried in patients without leprosy.[39] Steroids are commonly used in leprosy to alleviate the pain, due to their action on inflammation; they are not, however, specific treatments for the neuropathic pain itself. Different classes of drugs such as tricyclic antidepressants therefore need to be tested.

The main weakness of the study was that the grading of the pain intensity was not done. We also did not systematically exclude potential other pathologies such as diabetes or post herpetic neuralgia. This should be done in future studies. Furthermore, the diagnosis of psychological morbidity was not based on clinical psychiatric evaluation but on GHQ-12 scores only. For the questionnaire, we used the Hindi translation, which often had to be simultaneously translated to Marathi, and which has not yet been validated, and this may therefore have altered the results.

### Conclusions

The study demonstrates a high prevalence of neuropathic pain in patients who have completed treatment for leprosy and are generally not accounted for in leprosy statistics. The main symptom was numbness (86.4%) followed by tingling (68.2%), which differs from the findings from other studies.

The DN4 is a useful and simple tool for the screening of neuropathic pain, which makes it appropriate for leprosy field work. Its high specificity in the study is important in its further application outside research environment, considering the chronic implications of neuropathic pain.

The relationship of neuropathic pain with psychological morbidity found in this study further increases the magnitude of the problem. It will be important to continue research in this field in order to attend the needs of these patients in terms of prevention, diagnosis and treatment.

## Supporting Information

Checklist S1 STROBE checklist
(0.09 MB DOC)
